# Network Analysis Reveals TNF as a Major Hub of Reactive Inflammation Following Spinal Cord Injury

**DOI:** 10.1038/s41598-018-37357-1

**Published:** 2019-01-30

**Authors:** Weiping Zhu, Xuning Chen, Le Ning, Kan Jin

**Affiliations:** Shanghai Institute of Applied Mathematics and Mechanics, Shanghai University, Shanghai, 200072 P. R. China

## Abstract

Spinal cord injury (SCI) leads to reactive inflammation and other harmful events that limit spinal cord regeneration. We propose an approach for studying the mechanisms at the levels of network topology, gene ontology, signaling pathways, and disease inference. We treated inflammatory mediators as toxic chemicals and retrieved the genes and interacting proteins associated with them via a set of biological medical databases and software. We identified >10,000 genes associated with SCI. Tumor necrosis factor (TNF) had the highest scores, and the top 30 were adopted as core data. In the core interacting protein network, TNF and other top 10 nodes were the major hubs. The core members were involved in cellular responses and metabolic processes, as components of the extracellular space and regions, in protein-binding and receptor-binding functions, as well as in the TNF signaling pathway. In addition, both seizures and SCI were highly associated with TNF levels; therefore, for achieving a better curative effect on SCI, TNF and other major hubs should be targeted together according to the theory of network intervention, rather than a single target such as TNF alone. Furthermore, certain drugs used to treat epilepsy could be used to treat SCI as adjuvants.

## Introduction

Spinal cord injury (SCI) is followed by a complex cascade of inflammatory events, such as reactive astrocytosis, which upregulates many genes^1,2^ and forms a glial scar^3–5^. This typically results in a permanent loss of neurological function below the injury level^3,4^. Considerable efforts have been made in research involving SCI-induced inflammatory cytokines, such as neurotoxic reactive astrocytes induced by secreting IL-1α, TNF, and C1q that leads to activation of microglia^2^, and those represent potential therapeutic targets^6–10^; however, it is largely unclear how the genes associated with SCI (GAS) interact and which among them play key roles. We consider that a network-based integration and bioinformatic analyses of the data available for genes/proteins associated with disease will reveal possible mechanisms for assessing the effects of GAS on SCI, leading to greater clarity. As is known^11^, biochemical events driven by electrostatic forces and involving hydrophobic effects are the physical contacts with high specificity through which the gene or protein interaction network is established. In a network of biomolecules, the nodes (vertices) indicate genes or proteins, whereas the links (edges) indicate their physical (direct) or functional (indirect) interactions^12^. Network analysis has demonstrated an efficient approach for modeling biological systems^12–14^. For example, it could reveal the molecular mechanisms of cancer^13–17^ and infer the diseases associated with environmental chemicals^18,19^. During the last decade, with the popularization of RNA sequencing (RNA-seq) technologies and the development of bioinformatics analyses, a wealth of data for constructing a biomolecule network has been available from public databases/resources, such as Online Mendelian Inheritance in Man (OMIM)^20^, Kyoto Encyclopedia of Genes and Genomes (KEGG)^21^, Search Tool for the Retrieval of Interacting Genes/Proteins (STRING)^22,23^, and Comparative Toxicogenomics Database (CTD)^24–26^. OMIM is an updated catalog of human genes and genetic disorders and traits based on selection and review of the published peer-reviewed biomedical literature and has become one of the databases of the National Center for Biotechnology Information (NCBI)^27^; KEGG is a collection of databases with genomes, diseases, drugs, and chemicals, featuring the products of relevant pathways in biology; STRING includes experimental data, computational prediction methods and public text collections in biology, and links to numerous sources, and is able to generate known and predicted protein–protein interactions (PPIs); and CTD curates data on chemical–gene/protein interactions, chemical–disease and gene–disease relationships from selected literature sources in a structured format, and controlled vocabularies and inference scores, and integrates these data with those from NCBI, OMIM, KEGG, and 8 other databases, and links all the interactions to the original publications to enable users to access the source data for specific details about corresponding experiments. The inflammatory cytokines associated with SCI are neurotoxins^2^, and the toxicogenomics approaches can be used to identify them. TNF was first discovered in 1968 as a cytotoxic factor induced by lymphocytes and was referred to as a lymphotoxin (LT)^28^; therefore, it might be favorable for us to use the CTD database for collecting the SCI-gene data, and the CTD in-house scoring system for screening that data. There are thousands of curated genes associated with SCI in CTD that are available for the required bioinformatics analyses^26^, such as in this study about the effects of GAS on SCI.

In an effort to assess the effects of GAS on SCI, we designed a network-based integration and bioinformatics analysis approach, incorporating the disease-gene toxicogenomics^26^, PPI networks^22,23^, and gene ontology (GO) enrichment analysis^29–32^ and disease inference^26^. First, GAS were retrieved from CTD. Subsequently, the protein interactions involved in GASs were integrated from STRING database^22,23^, and visualized via Cytoscape^33–35^, a popular, open source bioinformatics software platform for network analysis. Finally, by using the interacting proteins, the functions and pathways associated with SCI were inferred. As a result, the most important as well as the top 30 interacting proteins were singled out; affected functions and pathways were identified; and diseases, including neurological and psychological disorders, were predicted, which provided better insight into the influence of GAS on SCI and related diseases. This analysis approach is also expected to be useful for studying neurotrophic factors and nerve growth factors involved in SCI and its consequences. Using biological data in system-level to study disease-gene associations is able to improve our current knowledge of disease relationships, leading to further improvements in disease diagnosis, prognosis and treatment.

## Material and Methods

### Genes/proteins associated with SCI (GAS)

GAS data were obtained from CTD^26^ by searching for genes involved in SCI, resulting in a list of 12,824 GAS or their protein products, which were then sorted by the CTD in-house “inference score” in descending order. The top 30 GAS (GAS30) with high scores (47.84-33.7) were taken as the core data in this study (i.e., in this context, the GAS30 represented the genes that were most closely associated with SCI). Then, Cytoscape (version3.4.0, 2016)^34^ and STRING (version 10.5)^23^ were conducted to query the protein-protein interactions of the GAS30. STRING is as an application (App, plugin) installed in Cytoscape. The data in STRING are weighted and integrated and a confidence score is calculated for all protein interactions according to the nature and quality of the supporting evidence. As a result, each of these interactions is assigned a confidence score between zero (no interaction) and one (high-confidence interaction), which indicates the probability that the interaction is authentic, given the available evidence. The default cutoff for confidence interactions is 0.4^18,19^. This study utilized this default value to screen PPIs and only the interactions whose confidence scores were >0.4 were considered for network analysis. Of the established PPI network of the GAS30, all nodes were from CTD and with CTD in-house inference scores of >33, and all edges were from STRING and with STRING in-house confidence scores of >0.4. Furthermore, the plugin NetworkAnalyzer^34^ in Cytoscape was used to visualize molecular interaction networks and integration with gene expression profiles and other state data.

### Enrichment analysis of gene ontology, pathway, and disease

Gene ontology (GO)^31,32^, a controlled vocabulary describing gene products and a useful resource for studying gene functions^36^, consists of three domains termed cellular components (CC), molecular functions (MFs), and biological processes (BP). Identifying enriched GO terms from a given gene list enables insight into the over-represented functions of genes^29^. Enrichment analysis of pathways and diseases is also an approach to the further understanding of the implicated pathways and diseases associated with SCI. Several web services such as the BinGO^37^ plugin of Cytoscape, OmicsBean^38^ and Set Analyzer^26^ of CTD can be employed for studying enriched GO terms, pathways and diseases, respectively. Among these services, the pathway-gene relationships for enrichment analysis are from the KEGG^21^ and REACTOME^39^ pathway databases, whereas the MEDIC disease vocabulary^24^ that combines the Medical Subject Headings (MeSH)^40^ and OMIM^20^ databases is used for analysis of enriched diseases. Briefly, we input the gene list of GAS30 respectively into BinGO or OmicsBean for GO term analysis; OmicsBean for pathways analysis; and the set analyzer of CTD for diseases analysis, while using a p-value of <0.05.

## Results

### PPI network analysis

**Figure 1:**
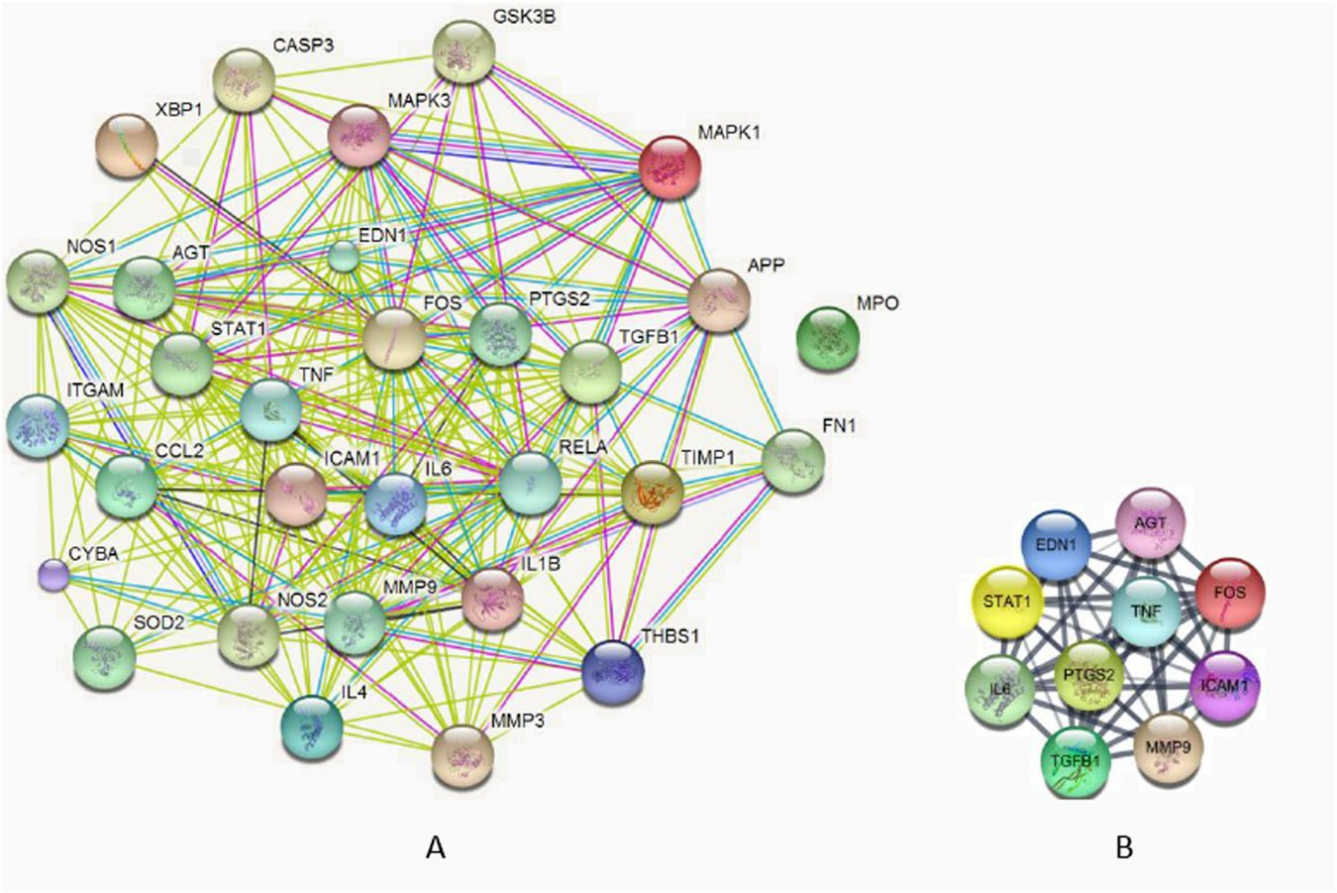
Protein–protein (PPI) networks. (**A**) The GAS30 network and (**B**) and GAS10 sub-network (**A**) presented in Table 2. Smaller nodes indicate the proteins whose three-dimensional structures were undetermined;larger nodes indicate the determined or predicted proteins. Colors of lines (edges) represent different interaction types.

A total of 12,824 genes were identified as associated with SCI using CTD as of December 12, 2017. Among these, the top 30 genes (GAS30) with CTD inference scores >33 are listed in Table 1. After inputting the GAS30 gene list into Cytoscape, assigning a link to STRING, and assuming that the interactions between the molecules were nondirectional and with interacting confidence scores of >0.4 in STRING, we obtained a PPI network for GAS30. Figure 1A shows a GAS30 PPI network that consists of 30 nodes and 232 edges. A node represents a molecule and an edge represents an interaction between two connected nodes. These two nodes are called neighbors. The fact that no edge is connected to a node such as myeloperoxidase (MPO) indicates that interactions between this node and others do not exist (at least their interacting confidence scores were ≤0.4) and it should be deleted from this network. The number of edges/neighbors related to a node is referred to as the degree of the node^35,41,42^. A node with a number of edges that greatly exceeds the average is referred to as hub and these play crucial roles in the network^43^. Therefore, molecules in the GAS30 network could be re-sorted by their node degrees. By use of NetworkAnalyzer^34^, a Cytoscape plugin for network topology analysis, the degrees of each node in the GAS30 network were calculated and the top 10 are listed in Table 2. Among these, the TNF node exhibited the greatest degree, and is termed a major hub. Furthermore, the top 10 interacting molecules become a sub-network of the GAS30 cohort, and are denoted as GAS10 and shown in Fig. 1B. The sub-network GAS10 comprised 10 nodes and 44 edges, leading to an extremely high clustering coefficient^41^ of 0.978 and an extremely small diameter^44^ of 2. That is, the 10 nodes were all major hubs and highly interconnected.

**Table 1:** The top 30 genes associated with spinal cord injury (SCI) from the Comparative Toxicogenomics Database (CTD) and genes associated with SCI (GAS30).

	Gene	Gene ID	Inference Score
1	MAPK1	5594	47.84
2	IL6	3569	47.13
3	AGT	183	45.15
4	CASP3	836	45.06
5	MAPK3	5595	44.58
6	CCL2	6347	44
7	TGFB1	7040	43.61
8	ITGAM	3684	43.11
9	MMP3	4314	41.76
10	EDN1	1906	41.69
11	IL1B	3553	41.52
12	FOS	2353	40.79
13	TNF	7124	40.13
14	TIMP1	7076	39.27
15	CYBA	1535	39
16	APP	351	37.77
17	ICAM1	3383	37.76
18	NOS2	4843	37.71
19	NOS1	4842	36.44
20	MMP9	4318	36.37
21	MPO	4353	36.37
22	XBP1	7494	36.3
23	FN1	2335	36.23
24	IL4	3565	36.04
25	GSK3B	2932	35.3
26	PTGS2	5743	35.1
27	RELA	5970	35.05
28	STAT1	6772	34.23
29	SOD2	6648	34.15
30	THBS1	7057	33.7

**Table 2:** The top 10 proteins associated with spinal cord injury (SCI) from Comparative Toxicogenomics Database (CTD), and genes associated with SCI (GAS30).

	Node	Full name of the node	Degree*
1	TNF	Tumor necrosis factor	25
2	FOS	FBJ murine osteosarcoma viral oncogene homolog	24
3	IL6	Interleukin 6 (interferon, beta 2)	22
4	PTGS2	Prostaglandin-endoperoxide synthase 2	22
5	TGFB1	Transforming growth factor, beta 1	22
6	ICAM1	Intercellular adhesion molecule 1	21
7	MMP9	Matrix metallopeptidase 9	20
8	STAT1	Signal transducer and activator of transcription 1	20
9	AGT	Angiotensinogen	19
10	EDN1	Endothelin 1	19

*Node degree was measured for GAS30 network members.

### Gene ontology (GO) analysis

**Figure 2:**
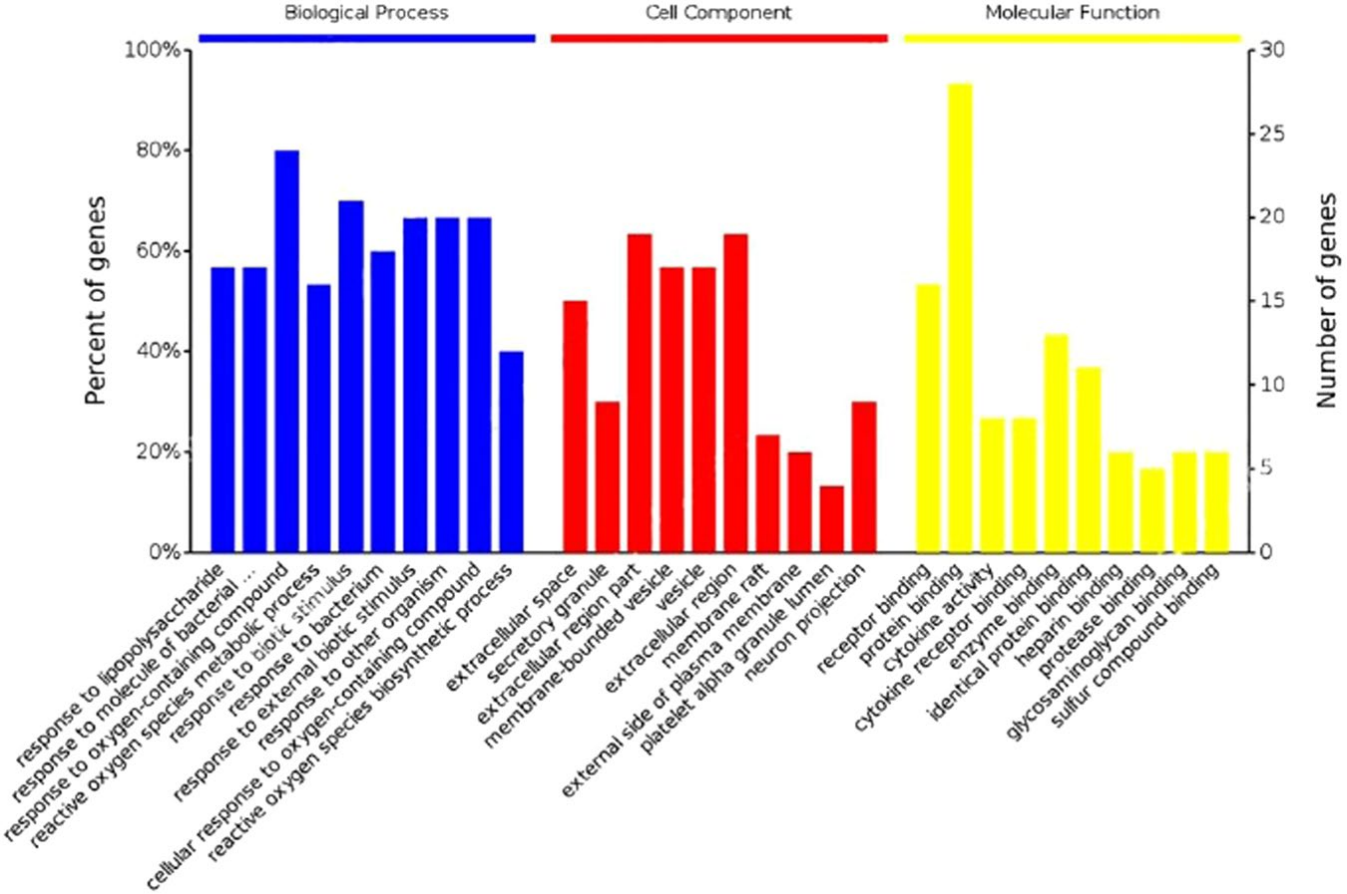
Histogram of top 10 enriched GO terms for GAS30 members.

GO term enrichment analyses of GAS30 proteins were conducted for biological processes (BPs), cellular components (CCs), and MFs using BinGO^37^ and OmicsBean^38^. Considering p < 0.05, there were 3,396, 210, and 251 terms for BPs, CCs, and MFs, respectively, enriched in GAS30. The top 10 terms for BPs, MFs, and CCs are listed in Table 3 and shown in Fig. 2.

**Table 3:** Top 10 enriched gene ontology (GO) terms with genes associated with SCI (GAS30) for biological processes (BPs), cellular components (CCs), and molecular functions (MFs).

	GO term name	GO term ID	P-value	Gene number
	**Biological Process (BP)**			
1	Response to lipopolysaccharide	GO:0032496	3.36e-29	17
2	Response to molecule of bacterial origin	GO:0002237	1.05e-28	17
3	Response to oxygen-containing compound	GO:1901700	1.39e-28	24
4	Reactive oxygen species metabolic process	GO:0072593	1.21e-27	16
5	Response to biotic stimulus	GO:0009607	2.03e-27	21
6	Response to bacterium	GO:0009607	6.74e-27	18
7	Response to external biotic stimulus	GO:0043207	6.27e-26	20
8	Response to other organism	GO:0051707	6.27e-26	20
9	Response to oxygen-containing compound	GO:1901701	3.35e-25	20
10	Reactive oxygen species biosynthetic process	GO:1903409	6.68e-25	12
	**Cellular Component(CC)**			
1	Extracellular space	GO:0005615	6.39e-13	15
2	Secretory granule	GO:0030141	1.02e-11	9
3	Extracellular region part	GO:0044421	3.61e-11	19
4	Membrane-bounded vesicle	GO:0031988	1.22e-09	17
5	Vesicle	GO:0031982	2.06e-09	17
6	Extracellular region	GO:0005576	2.37e-09	19
7	Membrane raft	GO:0045121	2.70e-09	7
8	External side of plasma membrane	GO:0009897	4.07e-08	6
9	Platelet alpha granule lumen	GO:0031093	1.76e-06	4
10	Neuron projection	GO:0043005	8.38e-08	9
	**Molecular function (MF)**			
1	Receptor binding	GO:0005102	2.02e-13	16
2	Protein binding	GO:0005515	4.57e-11	28
3	Cytokine activity	GO:0005125	5.05e-11	8
4	Cytokine receptor binding	GO:0005126	1.81e-10	8
5	Enzyme binding	GO:0019899	1.38e-09	13
6	Identical protein binding	GO:0042802	2.04e-09	11
7	Heparin binding	GO:0008201	5.70e-09	6
8	Protease binding	GO:0002020	2.16e-08	5
9	Glycosaminoglycan binding	GO:0005539	6.15e-08	6
10	Sulfur compound binding	GO:1901681	8.50e-08	6

The enriched BP analysis revealed that GAS30 could interfere with cellular responses and metabolic processes. Specifically, the process of responding to lipopolysaccharide involved 57% of GAS30 members (Fig. 2) and was promoted to the highest GO level in a significant p-value (Table 3). Lipopolysaccharide is a cell wall component of gram-negative bacteria, and is a type of endotoxin^45^ that is released only when bacterial cells are destroyed or when using an artificial method to kill the microorganisms. Considerable evidence has revealed the influence of lipopolysaccharides on central nervous system (CNS) diseases. For example, lipopolysaccharides can cause learning and memory disorders in rats subsequent to CNS inflammatory responses^46,47^, which positively supports the outcome of our GO term enrichment analysis for BPs.

**Figure 3:**
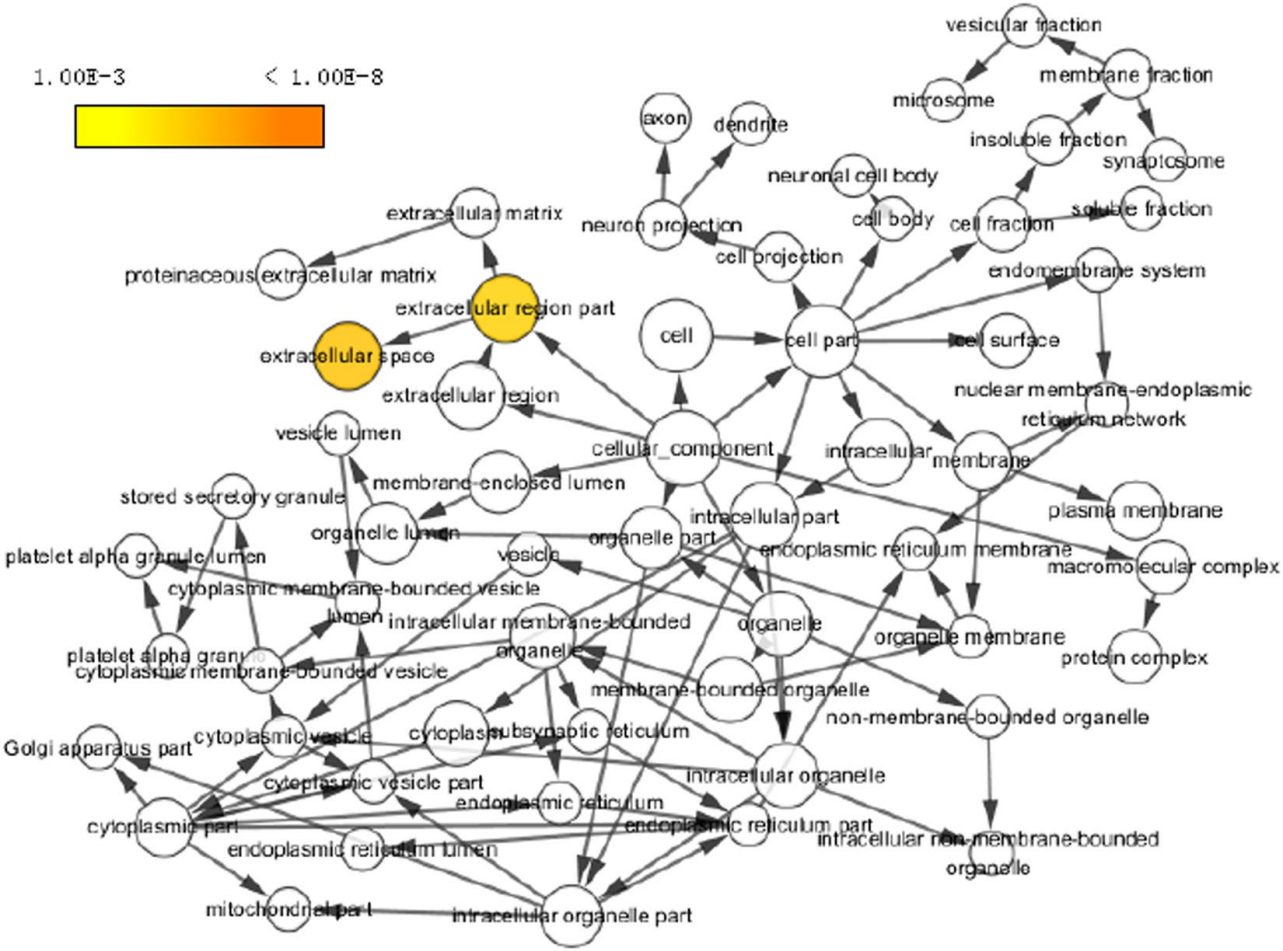
Hierarchical GO tree for cellular components enriched inGAS30. Considering a p-value of <0.05, the circle sizes are proportional to the number of genes included in each. A dark color indicates a highly significant p-value and a high degree of enrichment. White circles represent nonenrichment.

The enriched GO terms for CCs of the interacting proteins were mostly related to the extracellular space components, in which the first two (i.e., extracellular space and extracellular region components), exhibited the most significant p-values in CCs (Table 3) and accounted for 50% and 63% of GAS30 members (Fig. 2), respectively. A hierarchical GO tree for CCs enriched in GAS30 is presented in Fig. 3.

**Figure 4:**
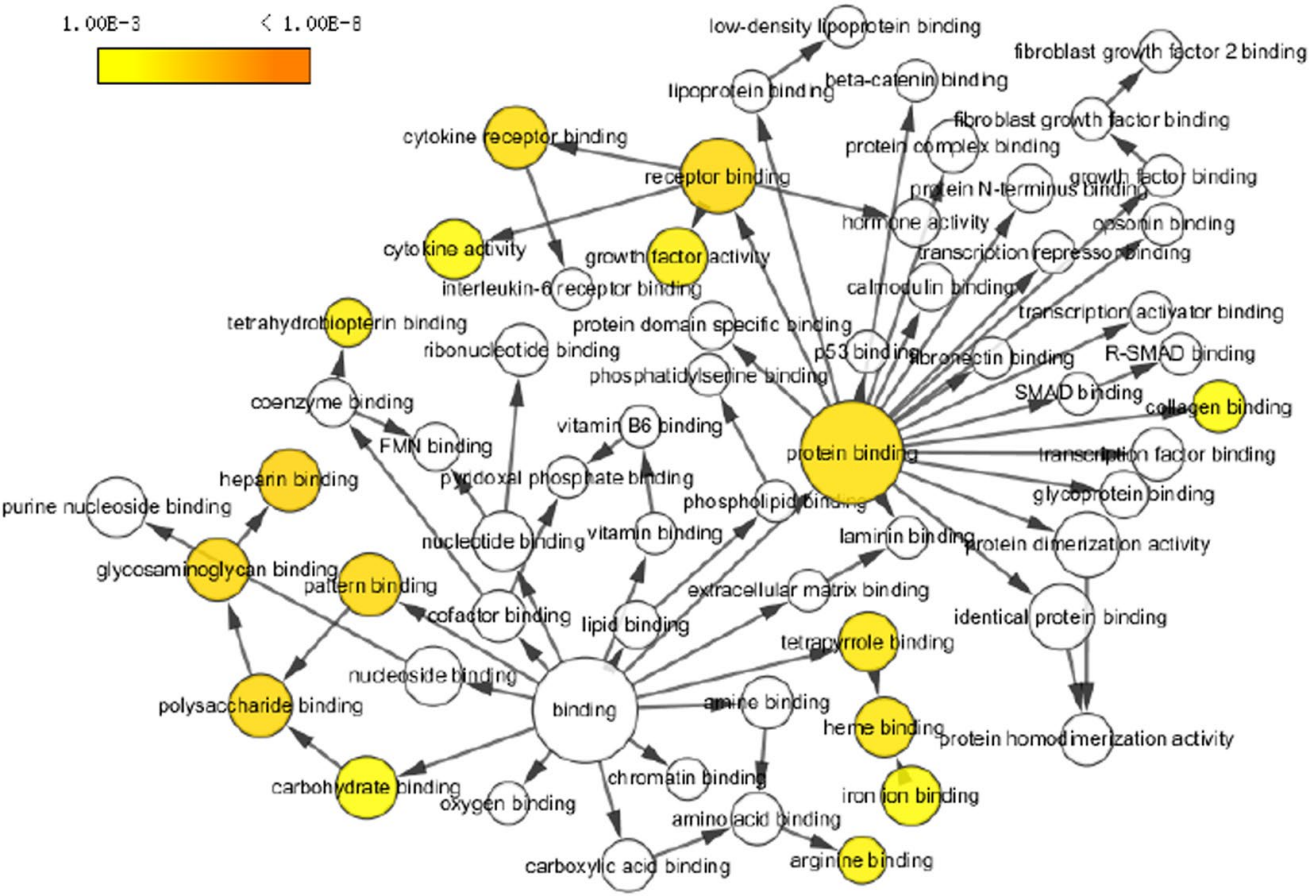
Hierarchical GO tree for molecular functions enriched in GAS30 members. Considering a p-value of <0.05, the circle sizes are proportional to the number of genes included in each. A dark color indicates a highly significant p-value and a high degree of enrichment. White circles represent nonenrichment.

The MFs influenced by the interacting GAS30 proteins were mostly related to the protein-binding and receptor-binding functions, according to the enriched GO terms. Notably, protein binding accounted for the highest percentage (93%) in GAS30 in all enriched GO terms as shown in Fig. 2. Figure 4 shows a hierarchical tree of important GO terms for MFs affected by the interacting GAS30 proteins.

### Pathway enrichment analysis

**Figure 5:**
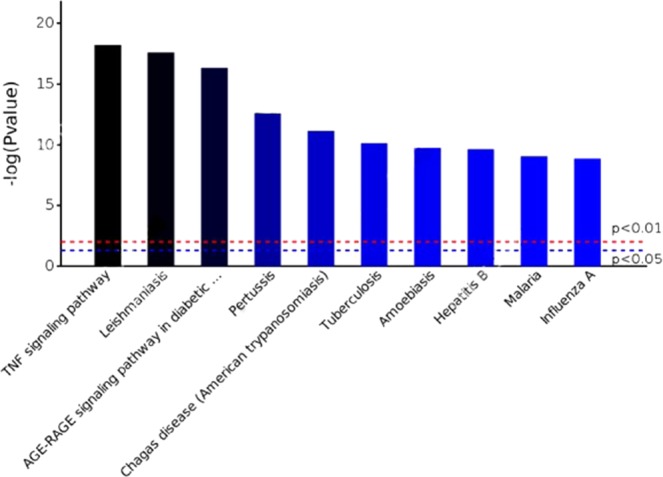
Histogram of the top 10 pathways involving GAS30 members. The horizontal axis indicates pathway names, whereas the vertical coordinates indicate the negative values of the log of the p-values: e.g., the horizontal dashed-lines in red and in blue have p-values equal to 0.01 and 0.05, respectively, and their vertical coordinates are equal to −log(0.01) = 2 and −log(0.05) = 1.3, respectively.

To further reveal the pathways affected by interacting GAS30 proteins, analyses were performed using OmicsBean^38^, a web service for processing biological data and with links to KEGG^21^ and other public databases. Following the instructions of OmicsBean, Table 4 and Fig. 5 were generated. The top 10 with the most significant p-values are listed in Table 4 and shown in Fig. 5. Specifically, the TNF signaling pathway was ranked at the top of the list, which accounted for 47% of GAS30 members.

**Table 4:** Top 10 pathways enriched with genes associated with SCI (GAS30).

	Pathway name	Pathway ID	P-value	Gene number
1	TNF signaling pathway	04668	2.53e-18	14
2	Leishmaniasis	05140	1.39e-18	13
3	AGE-RAGE signaling pathway in diabetic complications	04933	4.69e-17	13
4	Pertussis	05133	2.51e-13	10
5	Chagas disease (American trypanosomiasis)	05142	7.32e-12	10
6	Tuberculosis	05152	7.39e-11	11
7	Amoebiasis	05146	1.79e-10	9
8	Hepatitis B	05161	2.22e-10	10
9	Malaria	05144	8.99e-10	7
10	Influenza A	05164	1.41e-09	10

### Nervous system diseases involving TNF inferred from CTD

The diseases associated with TNF were inferred using the toxicogenomics analyses of CTD, which yielded 570 nervous system diseases associated with TNF. The top 30 diseases sorted by CTD in-house inference scores are listed in Table 5. Notably, among these, seizures had the highest score (No. 1 in Table 5).

**Table 5:** Top nervous system diseases associated with tumor necrosis factor (TNF) from the Comparative Toxicogenomics Database (CTD).

	Disease Name	Disease ID	Inference Score
1	Seizures	MESH:D012640	380.98
2	Memory disorders	MESH:D008569	374.18
3	Learning disorders	MESH:D007859	334.94
4	Hyperalgesia	MESH:D006930	334.94
5	Nervous system diseases	MESH:D009422	279.82
6	Muscular Diseases	MESH:D009135	250.16
7	Neurotoxicity Syndromes	MESH:D020258	248.65
8	Movement Disorders	MESH:D009069	235.38
9	Brain Diseases	MESH:D001927	234.22
10	Peripheral nervous system diseases	MESH:D010523	216.00
11	Tremor	MESH:D014202	205.22
12	Neurobehavioral manifestations	MESH:D019954	200.11
13	Coma	MESH:D003128	191.74
14	Brain injuries	MESH:D001930	188.08
15	Muscle Weakness	MESH:D018908	181.50
16	Vision Disorders	MESH:D014786	180.61
17	Confusion	MESH:D003221	179.57
18	Ataxia	MESH:D001259	176.91
19	Paresthesia	MESH:D010292	173.00
20	Central Nervous System Diseases	MESH:D002493	154.86
21	Brain Ischemia	MESH:D002545	151.31
22	Delirium	MESH:D003693	149.89
23	Hallucinations	MESH:D006212	146.49
24	Hyperkinesis	MESH:D006948	145.99
25	Neural tube defects	MESH:D009436	145.79
26	Stroke	MESH:D020521	143.60
27	status epilepticus	MESH:D013226	142.91
28	Dyskinesia, drug-induced	MESH:D004409	141.42
29	Catalepsy	MESH:D002375	141.34
30	Myoclonus	MESH:D009207	135.34

## Discussion

We considered SCI-induced inflammatory mediators as a type of toxin that inhibits the regeneration of injured tissue/cells, and we identified the associated genes and interacting proteins from known biological medical databases, CTD^26^, STRING^23^, and others, and chose the top 30 genes/proteins, GAS30, that were useful for studying their effects on SCI at the levels of network topology, GO, signaling pathways, and disease inference to provide a new visual angle for finding potential methods by which SCI intervenes.

According to this study, more than 10,000 genes associated with SCI and TNF achieved the highest score. TNF, FOS, IL6, and seven other of the top 10 nodes (Table 2) were the major hubs and highly interconnected in the GAS30 PPI network that were identified using CTD, STRING, and related databases. From the perspective of network topology^48^, such a network allows for a fault-tolerant behavior for which, if a hub-failure occurs, the network will generally not lose its connectedness because of the remaining hubs that will rapidly replace the failing hub. This suggests that although TNF negatively affected SCI repair, all other major hubs, such as FOS IL6, should be targeted simultaneously in the future for the development of new therapeutic approaches, rather than aiming at individual specific genes, one at a time, which might achieve better curative effects.

Furthermore, GAS30 members interfered mainly with cellular responses and metabolic processes, extracellular space and extracellular region components, protein-binding and receptor-binding functions, and TNF signaling pathways as identified by GO and pathway enrichment analyses. Notably, the TNF signaling pathways were promoted to the highest enriched level of GAS30 members and had the most significant p-value (Table 4). Although considerable evidence has revealed the influence of TNF as an inducer of inflammatory cytokines after SCI^7–9^ (e.g., neurotoxic reactive astrocytes induced by activation of microglia through secreting Il-1α, TNF, and C1q^2^), greater attention should be paid to TNF in the future and consider it to be a major signaling pathway and its use as a crucial and potential therapeutic target for SCI repair.

In addition, seizures were highly associated with TNF by CTD disease inference (Table 5). Clinically, seizures might occur after traumatic brain injury, and interestingly, repeated seizures might develop into post-traumatic epilepsy^49–51^. Seizures were also observed following SCIs^52,53^. More interestingly, the antiepileptic drug valproate was used as a supplement in stem cell transplantation for a mouse model of SCI, which dramatically enhanced the restoration of hindlimb function^54^. These suggest that certain drugs used to treat epilepsy could be employed as adjuvants in SCI treatment; however, these observations and suggestions were not directly linked to TNF by the original researchers, and the mechanisms proposed are unclear. Therefore, TNF, which is the most important hub identified in this study, could be further connected to the aforementioned findings and would be a direction for future SCI studies.

There are other aspects of this study that must be mentioned. In addition to CTD, the genes/proteins associated with SCI or other diseases that were searched to construct the PPI networks and subsequent bioinformatics analyses could be from OMIM^20,55^ or other publicly available databases^56^, which would generate similar results because nearly all public databases are interconnected to the Internet; therefore, the primary data mostly overlap with each other. As such, CTD includes OMIM and 10 other databases^26^. We used CTD in this study for its full name (Comparative Toxicogenomics Database) as well as its functions that matched our requirements for treating inflammatory cytokines in SCI as a type of neurotoxin. In recent years, disease genome sequencing and other high-throughput studies of disease genomes have generated many notable discoveries^17^. Direct data on disease-genes are commonly derived from RNA-seq because it is superior to other high-throughput technologies, such as microarray in accuracy, dynamic range, and differential expression detection, and has nearly completely replaced microarray for conducting genetic tests. The entries curated in OMIM have referenced^57–60^ the results from RNA-seq, and NCBI^27^ online accepts RNA-seq data and shares it with other databases and researchers. In addition, the data in references^2,5,8,9,^^13–17^ in this study were primarily from RNA-seq. Furthermore, a combination^61^ of using RNA-seq approach with PPI network analysis generated that TNF had the largest number of connected edges in the PPI network for contusive SCI in a mouse model, and the top ranked genes in the SCI gene list overlapped considerable with ours, supporting our current study; however, our present method hardly describes the dynamic effects of GAS on SCI. On the other hand, notably, because of its pleiotropic role, TNF shows, for example, a positive effect on regulatory T cells^62^ and prevents neurons from death/apoptosis by activating NF-κB^63,64^; therefore, suppressing TNF overexpression might not be a desirable intervention for SCI therapy, and this needs to be observed further.

Furthermore, to more effectively predict the SCI drug targets, the patient-specific signaling networks for reactive inflammation from SCI could be constructed using the concept of “SCI hallmarks” based on individual genomic data and on regulatory functions, just as the signaling networks of “cancer hallmarks”^13–17^ have been developed and substantially used for revealing molecular mechanisms of cancers and drug targets. This proposed approach to analyses is also expected to be useful for studying neurotrophic factors and nerve growth factors after SCIs.

With a constantly expanding repertoire of techniques, including RNA-seq, together with new information on genes and proteins, the current results will have more possibilities for examination and modification and will advance the current approaches to SCI analysis.

## Data Availability

All data generated or analyzed during this study are included in this published article.
